# Contrasting Patterns of Local Adaptation and Adaptive Potential Under Climate Change for Old‐Growth and Planted Stands of Norway Spruce (*Picea abies*)

**DOI:** 10.1111/eva.70217

**Published:** 2026-03-12

**Authors:** Helena Eklöf, Carolina Bernhardsson, Pär K. Ingvarsson

**Affiliations:** ^1^ Department of Ecology and Environmental Science, Umeå Plant Science Centre Umeå University Umeå Sweden; ^2^ Department of Organismal Biology, Evolutionary Biology Centre Uppsala University Uppsala Sweden; ^3^ Department of Plant Biology, Linnean Centre for Plant Biology Swedish University of Agricultural Sciences Uppsala Sweden

**Keywords:** forest regeneration, forestry, genetic differentiation, genetic diversity, Norway spruce

## Abstract

Genetic diversity is a key prerequisite for adaptation to changing environments. Maintaining genetic diversity in forest trees is crucial amid climate change, given their long generation times. Forest management practices can affect the genetic diversity of forest ecosystems through selective felling or reforestation strategies following harvests. To assess how managed forests respond to climate‐driven changes, we investigated patterns of genetic diversity and local adaptation by contrasting old‐growth and recently planted stands of Norway spruce ( *Picea abies* ). We assess both neutral and adaptive genetic variation by sequencing pooled samples collected from 45 first stands across northern Sweden. Our results reveal no significant differences in overall genetic diversity between natural and planted populations, indicating that current forest management practices have not substantially reduced genetic variation. Analyses of adaptive variation demonstrate strong signatures of local adaptation in old‐growth populations, with clear correlations between genetic and environmental distances. In contrast, planted stands show weaker adaptive signals and are also at greater risk of non‐adaptiveness under future climate scenarios. While current forest management practices preserve much of the neutral genetic diversity necessary for long‐term forest health, our findings highlight the importance of conserving and promoting adaptive genetic variation available in old‐growth stands to ensure resilience against ongoing climate change.

## 1 Introduction

Forest management aims to increase ecosystem benefits and services over those expected from unmanaged forests (Andersson et al. 2017). Management practices have been shown to affect forest diversity, from the genetic level to populations and ecosystems (Aravanopoulos 2018; Lefèvre 2004). Ongoing climate change is also expected to affect forest ecosystems, and even though most forests have extensive adaptive capabilities, adaptive forestry practices are needed in managed forests to respond to climate‐driven changes (Aitken et al. 2008; Alberto et al. 2013; Aravanopoulos 2018; Fady et al. 2020). One of the most important factors to consider when managing forests for climate change is preserving genetic diversity, as high levels of genetic diversity are essential for maintaining the adaptive capacity of forest populations (Aitken et al. 2008; Lefèvre et al. 2013). Forest management and silviculture can significantly affect genotypic and phenotypic variation in forests, for example, by selecting trees for felling or by choosing reforestation strategies following harvests. Several reforestation methods are used in contemporary commercial forestry, including leaving natural seed trees, direct sowing of seeds, or planting pre‐established seedlings (Aravanopoulos 2018; Nilsson et al. 2010). Planting genetically improved seedlings from nurseries is the prevailing method in many parts of the world, as it accelerates reforestation, allows for more even stands, and reduces the need for thinning. For example, 84% of all reforestation in Sweden is achieved through seedling plantations, most of which are obtained from seed orchards made up of genetically improved material. Natural regeneration accounts for an additional 10%, and direct seeding and no measures taken (where the regeneration methods are unknown) account for the remaining fraction of new stand establishments in Sweden (Black‐Samuelsson et al. 2020). Although large‐scale reforestation has been ongoing since the mid‐20th century in many parts of the world, it is unclear to what extent such practices, especially in combination with genetically improved material from existing breeding programmes, alter genetic diversity in planted forest stands. Using improved seeds from a seed orchard with a relatively small number of unique, genetically improved trees could reduce the effective population size of newly planted stands and, hence, limit genetic diversity (Ingvarsson and Dahlberg 2019; Savolainen and Kärkkäinen 1992). The recommended optimal number of unique genotypes included in a seed orchard, to maximise the estimated benefit, has been estimated to be 16 (Lindgren and Prescher 2005), assuming unrelated parent trees with normal fertility variation and that any pollen contamination is derived from unrelated sources (Lindgren and Prescher 2005).

The genetic diversity in Norway spruce seed orchard crops has been investigated by Sønstebø et al. (2018) using 11 microsatellites. They compared two seed orchards, each with 25 or 60 parent trees, and seed crops collected from semi‐natural or unmanaged forests consisting of 25 and 60 parent trees, with seed crops collected from semi‐natural or unmanaged forests from the same regions (Sønstebø et al. 2018). They showed that genetic diversity in orchard seed crops was slightly lower than in seed crops collected from semi‐natural or unmanaged forests (Sønstebø et al. 2018). Similarly, nine breeding populations from northern and central Sweden were examined using 15 simple sequence repeat (SSR) markers, and the results showed high genetic diversity within populations, low genetic differentiation between populations, and low levels of inbreeding and relatedness (Androsiuk et al. 2013). Finally, Verbylaitė et al. (2017) evaluated genetic diversity in self‐regenerated populations of Norway spruce and Scots pine in Lithuania. They observed high genetic diversity and low genetic differentiation between maternal trees and regenerating seedlings, suggesting that self‐regenerating populations can provide sufficient genetic diversity to ensure ecologically and evolutionarily sound stands (Verbylaitė et al. 2017).

Several studies have also compared genetic diversity between managed and natural stands of different conifer species to assess these possible concerns. Bergmann and Ruetz (1991) used isozyme markers and found no significant difference in gene diversity between forest samples and seed orchard clones in a German forest district. They did, however, observe a difference in average heterozygosity, suggesting slight differences in genetic diversity between the two sample types. Maghuly et al. (2006) conducted a study in Austria using microsatellites derived from mitochondrial, chloroplast, and nuclear DNA to compare two age groups (6–10 years and 70–100 years) from three subpopulations (each from a different elevation). The nuclear SSR markers showed slightly higher genetic variation within populations of both age groups than genetic differentiation among subpopulations. Similarly, Ruņģis et al. (2019) used 11 SSR markers to compare naturally regenerated Norway spruce ( *Picea abies* ) populations to progenies derived from two different seed orchards in Latvia. Ruņģis et al. (2019) found that the total and effective number of alleles, average number of alleles, average gene diversity and average allelic richness were higher in the naturally regenerated forests compared to the seed orchards. Genetic diversity indicators were similar across all populations, and genetic diversity in progeny from seed orchards was comparable to that in naturally regenerated forests. Finally, in Scots pine ( *Pinus sylvestris* ), nuclear‐ and chloroplast‐derived microsatellites were used to compare genetic diversity among natural forests, seed‐tree‐generated forests, and stands planted with genetically improved seedlings from three regions in Sweden (Garcia‐Gíl et al. 2015). The results showed that reforestation methods had no effect on nuclear or chloroplast genetic diversity. However, the number of effective alleles and total gene diversity in chloroplast markers were significantly higher in stands from seed trees than in natural forests and seedling‐planting stands (Garcia‐Gíl et al. 2015). Previous studies assessing genetic diversity in natural and managed stands of forest trees (Bergmann and Ruetz 1991; Garcia‐Gíl et al. 2015; Maghuly et al. 2006; Ruņģis et al. 2019; Sønstebø et al. 2018) have been based on either low‐resolution markers (e.g., allozymes) or have relied on a small number of high‐resolution markers (e.g., microsatellites) that have limited the statistical power for detecting systematic differences in genetic diversity or differentiation. Such markers are also generally not considered directly involved in controlling adaptive traits and therefore convey little information about the present and future adaptive potential of different forest stands.

Studies of genetic diversity and differentiation in many forest trees have observed that genetic differentiation among local populations is low, consistent with the extensive and homogenising effects of gene flow (Neale and Ingvarsson 2008; Savolainen and Pyhäjärvi 2007). However, genetic differentiation at loci underlying adaptive traits is expected to be greater, driven by the diversifying effects of local selection and possibly reduced gene flow (Savolainen et al. 2007, 2013). Earlier studies have confirmed that patterns of adaptive variation are often strikingly different from those seen in neutral genetic variation (Le Corre and Kremer 2003; Savolainen et al. 2013). Since natural selection favours alleles that enhance adaptation to local growing conditions, it often leads to greater genetic differentiation among populations for traits that contribute to local adaptation (Savolainen et al. 2007, 2013). In most forest trees, such local adaptation persists despite high levels of gene flow that continuously introduces potentially maladaptive genetic variation, generating clinal variation in many adaptive traits across environmental gradients (Kremer et al. 2012; Savolainen et al. 2007). Such clinal variation is also ubiquitous in Norway spruce (Chen et al. 2012; Kapeller et al. 2017; Suvanto et al. 2016).

Modern forest tree breeding programmes typically include climate adaptation as a primary breeding objective (Cortés et al. 2020; Holliday et al. 2017; Isabel et al. 2020). However, to date, few studies have addressed how current forest management practices alter adaptive genetic variation and local adaptation in situ. Norway spruce ( *Picea abies* (L.) Karst.) is one of the most important conifer species in Europe, with a distribution range extending from the west coast of Norway to mainland Russia in northern Europe and across the Alps, the Carpathians and the Balkans in central Europe (Giesecke and Bennett 2004). In 2013, there were around 3.5 billion cubic metres of standing forest on productive forest land in Sweden, with 41% of Norway spruce and 39.1% of Scots pine (Black‐Samuelsson et al. 2020). Norway spruce is characterised by large population sizes and low mutation rates, which, together with its outcrossing, wind‐pollinated breeding system, lead to high genetic diversity within populations and low differentiation among populations (Burczyk et al. 2004). Breeding of Norway spruce in Sweden began in the 1950s by selecting plus trees (i.e., trees with phenotypically desirable qualities, such as straight trunks, free of damage, and high quality) from natural forests. Today, breeding populations consist of trees of both Swedish and foreign origin, which are included to improve genetic gain and are extensively evaluated using field testing. Material from breeding populations has been used to establish commercial seed orchards to maximise genetic gains in value and volume production (Lindgren et al. 2007).

In this study, we assess genetic diversity in a selection of old‐growth stands of Norway spruce in northern Sweden that have not been logged for at least 150 years. We contrast the natural, old‐growth stands with similar data obtained from recently planted stands (< 25 years) located near the old‐growth populations. Our goal is to assess how patterns of genetic diversity differ between the two stand types and to identify genetic variation associated with local climate. We also evaluate the relative importance of isolation by distance and isolation by environment (Wang and Bradburd 2014) for the different types of stands. Finally, we use climate modelling data to assess the risk of maladaptation of different stand types to projected climate changes at the end of the 21st century.

## 2 Method

### 2.1 Sampling

**FIGURE 1:**
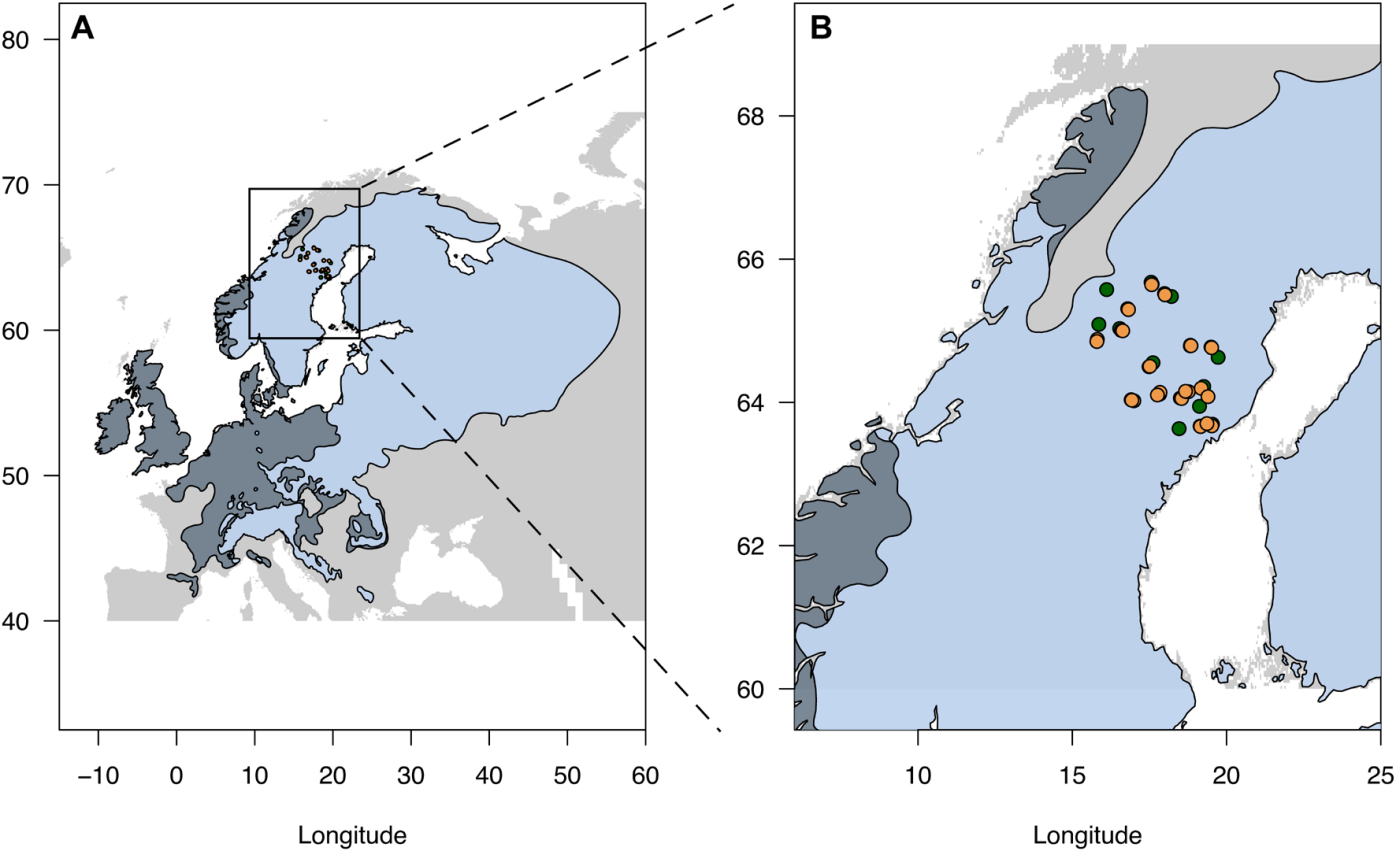
(A) The natural (light blue) and introduced (dark slate) distribution range of *P. abies* in Europe. (B) Close‐up of northern Sweden with the sample sites highlighted in green for old and tan for planted populations.

We collaborated with Länsstyrelsen Västerbotten to identify and obtain coordinates for old‐growth forest stands in the counties of Västerbotten and Västernorrland in northern Sweden (Figure 1, Table S1). We selected 15 stands that have not been logged for at least 150 years. These stands ranged from the Baltic coast in the east to the Scandinavian mountains in the west, capturing the east‐to‐west distribution of Norway spruce ( *Picea abies* ) in northern Sweden (Figure 1, Table S1). Near each old‐growth stand, we identified two newly planted forest stands (planted < 25 years ago). We obtained stand coordinates, information on the origin of planting material and date of replanting, where available, from two forest companies active in the region, SCA Skog AB (11 stands) and Sveaskog (19 stands). For the Sveaskog stands, we obtained the actual replanting year, whereas for the SCA stands, we obtained the year of clear‐cutting. Replanting after clear‐cutting usually occurs within 1–2 years, so we arbitrarily set the planting date to one year after clear‐cutting. Unfortunately, we were unable to determine whether the trees planted at each site were derived from Swedish or foreign seed orchards or from collections of natural populations within Sweden or elsewhere. However, statistics on seedling plantations from the Swedish Forest Agency (https://www.skogsstyrelsen.se/en/statistics/) suggest that 65%–80% of planted Norway spruce seedlings are derived from Swedish seed orchards and an additional 10%–13% are derived from foreign seed orchards.

In June 2017, we visited all 15 old and 30 young, planted stands, and sampled newly flushed buds from 50 randomly selected trees using transect sampling to obtain material from 2250 trees. All samples were kept on ice during field sampling, then returned to the lab and stored at −80°C until DNA extraction. From the 50 samples per stand, we randomly selected 30, resulting in a total of 1350 trees used for DNA extraction.

### 2.2 DNA Extraction, Sequencing and SNP Calling

Population genetic analyses rely on accurately estimating allele frequencies from population samples. Sequencing pooled samples of individuals (Pool‐Seq) has been shown to provide more accurate allele frequency estimates than sequencing individuals, as more individuals can be assessed with a Pool‐Seq approach and usually at only a fraction of the cost of sequencing separate individuals (Schlötterer et al. 2014). To genotype as many trees and stands as possible, we adopted a Pool‐Seq strategy for our samples.

DNA was extracted from all samples using an Omega Bio‐tek E‐Z 96 plant kit (OMEGA Bio‐Tek), and DNA concentrations were measured on a Qubit (ThermoFisher Scientific). High‐concentration samples were diluted, and 30 samples from each stand were pooled in equimolar concentrations by using 200 ng of DNA from each sample. Each pool was divided into eight tubes (750 ng of DNA per tube) to maintain a low reaction volume. The GBS library preparation method is described in detail by Pan et al. (2015), with minor modifications outlined below.

DNA digestion and ligation were performed in 50 μl reagent systems using the restriction enzyme *Pst*I (New England BioLab, Woburn, MA, USA). Adaptors were ligated to each of the 45 DNA stand pools using five unique barcodes. Five stand pools with unique barcodes were further pooled to create a super‐pool. The nine super‐pools (5 stand pools per super‐pool) were purified using a QIAquick PCR purification Kit (Qiagen, Hilden, Germany), and DNA concentrations were measured using a Qubit fluorometer (ThermoFisher Scientific). All nine super‐pools were amplified by PCR in 12 50 μL reactions per pool, then purified. Size selection was made with the E‐gel Size‐Select II pre‐cast gel (ThermoFisher Scientific), using a total of 100 μl sample from each super‐pool. Targeting fragments in the range 350–450 bp (accounting for 125–132 bp barcodes and sequencing adapters), the gel was run for approximately 20 min before the desired fragment size was excised from the gel. The gel was cut, and DNA was extracted using a QIAquick Gel Extraction Kit (Qiagen, Hilden, Germany), resulting in nine unique super‐pool libraries. Each library was quantified on a Qubit (ThermoFisher Scientific), and pair‐end sequencing (2 × 150 bp) was performed on an Illumina HiSeqX by Novogene Europe. Each super‐pool was sequenced individually on one HiSeq X lane, yielding > 120 Gbp raw sequencing data per super‐pool.

The raw sequencing data were quality checked using FastQC v0.11.8 (https://www.bioinformatics.babraham.ac.uk/projects/fastqc/), and sequencing adaptors were trimmed using Trimmomatic v0.36 (Bolger et al. 2014). The sequence data from each super‐pool library were demultiplexed using the process_radtags routine from Stacks v2.2 (Catchen et al. 2011) to extract reads for the individual stand pools. All sequencing reads were mapped against the *P. abies* v1.0 genome (Nystedt et al. 2013) using BWA‐MEM with default parameters (Li 2013). For SNP calling, we used samtools v1.14 (Li et al. 2009) to create an mpileup file from all 45 individual BAM files and SNPs were then called from this file using VarScan v2.4.1 (Koboldt et al. 2012).

### 2.3 Data Analysis

All SNPs, in VCF format, were read into R using the vcf2pooldata function from the poolfstat package (Gautier et al. 2022; Hivert et al. 2018). The SNP data were filtered to include only SNPs with a minimum coverage of 60 reads, corresponding to 1× per haplotype in the pool. We further required the coverage of all pools to fall within the 0.1 to 99.9 quantile coverage thresholds and have a minimum allele frequency of 0.008. All further analyses were performed in R using the poolfstat package. Genetic differentiation measured as Wright's fixation index, *F*_ST_, was calculated using methods outlined in Hivert et al. (2018). The geographic distance between stands was estimated from their respective latitude and longitude coordinates using the Haversine formula, which calculates the shortest distance, also known as the ‘great‐circle‐distance’, between two points using the distHaversine function from the geosphere package in R. To assess the genetic similarity between stands within a single locality, that is, one old‐growth and the two corresponding newly planted stands, we employed the outgroup *f*_3_ statistics (Patterson et al. 2012). Outgroup *f*_3_ statistics are a special case of how *f*_3_ statistics are commonly used. Instead of a target population and two possible source populations, as is used in calculating ordinary *f*_3_ statistics for admixture, the outgroup version uses two source populations and one outgroup (Gautier et al. 2022; Patterson et al. 2012). The test estimates genetic similarity, also known as ‘shared genetic drift’, between the source populations and the outgroup (Gautier et al. 2022). We calculated outgroup *f*_3_ tests using all combinations of old and planted stands within localities by cycling through which stand type was assigned as the outgroup. In this type of analysis, higher *f*_3_ statistics indicate that the two source populations are more genetically similar relative to the outgroup.

### 2.4 Current and Future Climate Data

Climate data for all stands were obtained from the geographic coordinates assigned to each stand during sampling using the ENVIREM database (http://envirem.github.io/, Title and Bemmels 2018) with a spatial resolution of 2.5 arcmin (~5 km). Climate data were obtained for either the current climate (1960–1990) or for the predicted climate in the year 2070 based on a representative future greenhouse gas concentration pathway (RCP4.5; Moss et al. 2008). The RCP4.5 data were constructed based on climate data from Worldclim (v1.4, Hijmans et al. 2005, http://worldclim.org/current), also at a 2.5 arcmin resolution, and converted to ENVIREM data format as previously described (Ingvarsson and Bernhardsson 2020). Due to high collinearity among variables in the ENVIREM data, we selected seven representative climate variables that describe the variation in temperature and aridity across the study region (Figure S1). The climate variables used in all further analyses are listed in Table 1 (Title and Bemmels 2018).

**TABLE 1:** Climate variables and abbreviations used.

Climate variable	Abbreviation	Brief description
Aridity Index Thornthwaite	AIT	Index of the degree of water deficit below water need
Climatic Moisture Index	CMT	A metric of relative wetness and aridity
Continentality	CONT	Average temperature of warmest month—average temperature of coldest month. Measured in units of °C.
Growing Deg Days 0C	DegD0	Sum of mean monthly temperature for months with mean temperature greater than 0°C multiplied by number of days. Measured in units of 10*°C.
Growing Deg Days 5C	DegD5	Sum of mean monthly temperature for months with mean temperature greater than 5°C multiplied by number of days. Measured in units of 10*°C.
Max Temp Coldest	MTC	The maximum temperature of the coldest month. Measured in units of 10*°C.
PET Coldest Quarter	PCQ	Mean monthly potential evapotranspiration (PET) of coldest quarter

To assess differences in climate between sites, we calculated the pairwise climate distance between sites using the Euclidean distance in multi‐dimensional climate space, $D_{\mathrm{ab}}$, where each dimension, *i*, corresponds to a normalised climate variable for sites *a* and *b* (Harris et al. 2025; Hubbard et al. 2025), as implemented in the vegdist function from the R‐package vegan (Oksanen et al. 2025):

$$D_{\mathrm{ab}}=\sqrt{\sum_{i}{\left(a_{i}-b_{i}\right)}^{2}}.$$

### 2.5 Gene–Environment Association Analyses

SNPs significantly associated with climate variables were identified in two ways. First, we selected SNPs based on the correlation between allele frequencies and the climate variable of interest across stands using the approach outlined by Fischer et al. (2013). Briefly, we generated 1,000,000 random allele‐frequency variables and paired each draw with a randomly selected climate variable to generate a null distribution of the correlation coefficient between allele frequencies and climate variables. We then used the 99.9% quantile of the resulting 1,000,000 correlation coefficients (i.e., *r* = 0.730) as our threshold to select SNPs deemed significantly associated with a particular climate variable. If an SNP‐environment correlation exceeded this threshold, the SNP locus was considered significantly associated with the environmental variable. We selected associated SNPs based on whether they were associated with a climate variable in old or planted stands separately. The second approach to identify outlier SNPs used latent factor mixed models (LFMM) (Caye et al. 2019; Frichot et al. 2013) as implemented in the lfmm package in R (https://CRAN.R‐project.org/package=lfmm). The main benefit of the LFMM method is that it can adjust gene–environment associations for underlying population structure, which the simple correlation method cannot do. Population stratification is a major issue in genetic association studies because it creates spurious associations (false positives) by confounding genetic data with ancestry rather than with the traits of interest (Sul et al. 2018; Vilhjálmsson and Nordborg 2013). To alleviate such confounding, we first used a principal component analysis to estimate population structure from the SNP data (Figure S2A). Based on the scree plot (Figure S2B), we selected four (*K* = 4) latent factors to correct for population structure in the data. These first four principal components from the population structure PCA were included as latent factors in LFMM to account for the underlying population structure of the data (Caye et al. 2019). Consistent with how we conducted the correlation tests, we separately estimated the associated SNPs for old and planted stands. Individual SNPs were deemed significantly associated with a climate variable if the associated *p*‐value was less than the Bonferroni‐corrected significance threshold of 1.05 × 10^−6^ (=0.05/47,552 SNPs).

Isolation by environment (IBE) is a pattern of population genetic structure where genetic differentiation increases with environmental differences, independent of geographic distance. It occurs when ecological factors, such as climate, reduce gene flow, thereby acting as barriers that promote local adaptation and divergence between populations (Wang and Bradburd 2014). To test for isolation by environment, we used partial Mantel tests to test for overall associations between genetic differentiation among stands and climate distance. This method has been successfully used in previous studies assessing associations between population differentiation and climate variables (e.g., Fischer et al. 2013; Hancock et al. 2011; Nosil et al. 2012). The method allows for comparing two pairwise distance matrices while controlling for the effect of a third. The dependent variable in all analyses was the pairwise *F*_ST_ matrix, calculated from outlier SNPs selected using either correlations or LFMM, as outlined above. The predictor variable was the ‘environmental distance’ between sites, calculated by the Euclidean distances between sites for the corresponding environmental variable using the vegdist function from the vegan package in R. The matrix used to control for background population structure was constructed from pairwise *F*_ST_ values calculated from all remaining SNPs after outlier SNPs were removed. Partial Mantel tests were run using Pearson's *r*, thus assuming a linear relationship between allele frequencies and environmental factors. The partial Mantel tests were implemented using the mantel.partial function from the vegan package in R.

Finally, we calculated the risk of non‐adaptedness (RONA) following Rellstab et al. (2016). For the RONA analyses, we compared the current climate at all sites with the expected climate derived from the RCP4.5 greenhouse gas concentration scenario (Moss et al. 2008). The RONA analyses estimate the average expected allele frequency shifts required for populations to track under future environmental conditions across all associated SNPs, with each locus weighted by the *R* ^2^ value of the allele frequency‐environment correlation (Pina‐Martins et al. 2019). The RONA estimations were based on all SNPs associated with the selected climate variables, either via simple correlations or through LFMM analyses. RONA calculations were performed separately for the old and planted stands. To run the RONA analyses, we used scripts obtained from Dryad for the publication by Dauphin et al. (2021) (https://datadryad.org/dataset/doi:10.5061/dryad.866t1g1pm).

## 3 Results

We generated over 1100 Gbp of sequencing data across the 45 sequence pools, with a median coverage of 1850× per site across the 1.1 Mb of the Norway spruce genome that our GBS analysis targeted (Table S2). After SNP calling and filtering, 47,552 SNPs were retained for all subsequent analyses.

**FIGURE 2:**
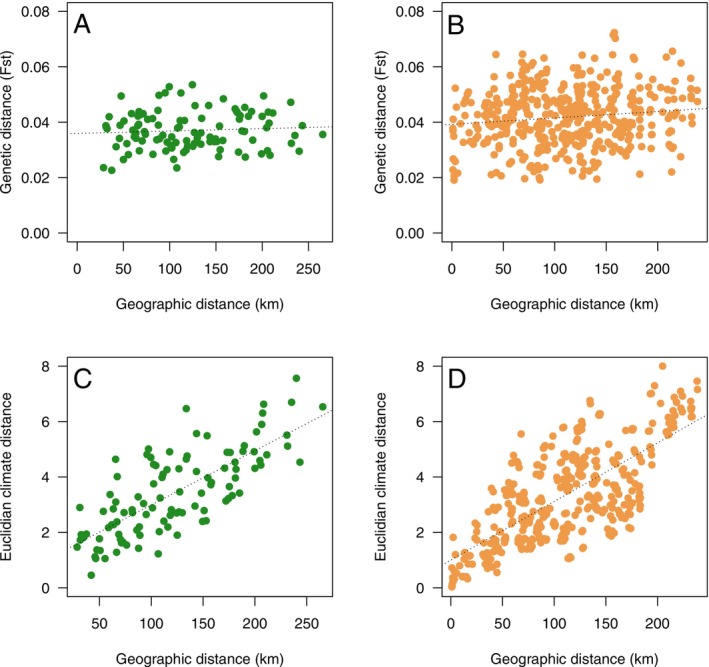
Isolation by distance for (A) old and (B) planted populations. Correlation between geographic and climate distance for (C) old and (D) planted populations. Dotted lines are the best‐fitting regression lines and are only used for visual guidance.

We used all SNP data from the complete set of individual pools to assess population structure in our collection of stands (Figure S2). We also calculated pairwise *F*_ST_ values between all stands. Using a Mantel test, we assessed the evidence for isolation by distance by calculating the correlation between pairwise population differentiation and the great‐circle distance between stands. Interestingly, we detected only weak isolation by distance in our study populations, which was significant only for the planted populations (old: *r* = 0.075, *p* = 0.254; planted: *r* = 0.127, *p* = 0.036; Figure 2A,B). For both sets of populations, we observe strong and significant associations between geographic and climate distance (Figure 2C,D).

**FIGURE 3:**
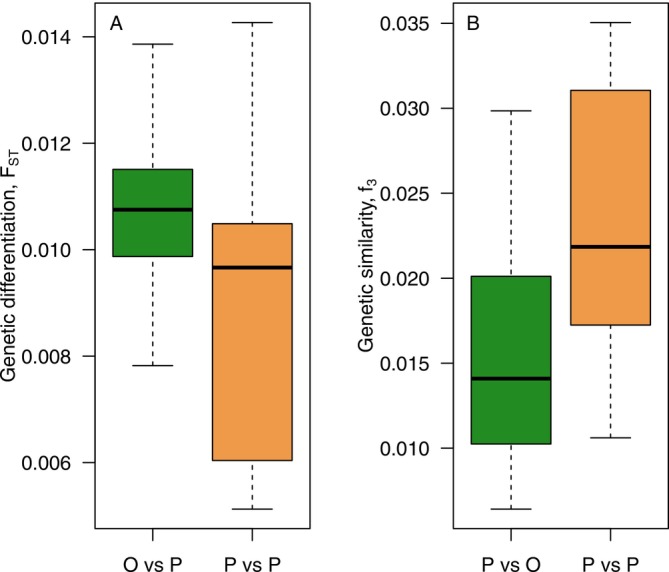
(A) Pairwise genetic differentiation and (B) pairwise genetic similarity (shared genetic drift, f3) between old and planted (O vs. P) or between planted populations (P vs. P) within each location.

When analysing different estimates of genetic diversity on a per‐stand basis, we did not observe any significant differences between old and planted stands for the number of invariant sites, the proportion of rare alleles (alleles with a frequency < 5%) or heterozygosity (Figure S2). Finally, we assessed pairwise genetic differentiation (*F*_ST_) and genetic similarity (outgroup *f*_3_) among the different stand types within each sample location. We observed a higher genetic differentiation between planted and old stands (Figure 3A) and a greater genetic similarity among the planted stands (Figure 3B).

We observe substantially more significant associations between genetic diversity and climate in the old‐growth stands when using simple allele‐frequency correlations with the climate variables (the average number of significant correlations for old‐growth and planted stands is 96 and 5, respectively; Table 2). We also observed strong, positive partial Mantel correlations for the old‐growth stands with all climate variables. At the same time, only AIT was significant for the planted stands (Mean partial Mantel *r* = 0.914 and *r* = 0.028 for old‐growth and planted stands, respectively, Table 2). These correlations remain strong for old‐growth stands under the projected climate for the RCP45 2070 scenario (mean partial Mantel *r =* 0.833), while no such correlations were observed in the planted stands (mean partial Mantel *r =* −0.011, Table 2).

**TABLE 2:** Associations and partial Mantel correlations for old‐growth and planted stands, using allele‐frequency correlations to select outlier loci.

Variable	Number of SNP associations		Partial Mantel			
			Current		RCP45 2070	
	Old	Planted	Old	Planted	Old	Planted
Aridity Index Thornthwaite	99	5	**0.895**	**0.156**	**0.883**	0.061
Climatic Moisture Index	81	0	**0.940**	−0.028	**0.939**	−0.030
Continentality	150	0	**0.951**	−0.152	**0.962**	−0.158
Growing Deg Days 0C	110	8	**0.909**	0.106	**0.792**	0.071
Growing Deg Days 5C	125	3	**0.893**	0.102	**0.454**	−0.016
Max Temp Coldest	39	2	**0.886**	−0.011	**0.873**	−0.030
PET Coldest Quarter	68	14	**0.925**	0.019	**0.930**	0.022

*Note:* Bold correlations are significant at *p* < 0.001.

We observed similar results when outlier SNPs were selected using LFMM, although the overall patterns were not as strong as those in analyses based on simple correlations, suggesting that the correlation analyses may be partly confounded by the underlying population structure. On average, 35 SNPs show significant associations with climate in old‐growth stands compared to only 3 in the planted stands (Table 3) in the LFMM data set (Table 3). The partial Mantel correlations based on the LFMM‐outlier SNPs for the old‐growth stands were also high and significant for all climate variables (mean partial Mantel *r* = 0.715). At the same time, only CONT was significant for the planted stands (mean partial Mantel *r* = 0.016). For the 2070 RCP45 climate scenario, all climate variables, except DegD5, remained significantly correlated for old‐growth stands (mean partial Mantel *r* = 0.645, Table 3). For the planted stands, none of the partial Mantel correlations was significant for this climate scenario (mean partial Mantel *r* = 0.008, Table 3).

**TABLE 3:** Associations and partial Mantel correlations for old‐growth and planted stands using LFMM to select outlier loci.

Variable	Number of SNP associations		Partial Mantel tests			
			Current		RCP45 2070	
	Old	Planted	Old	Planted	Old	Planted
Aridity Index Thornthwaite	25	0	**0.760**	0.073	**0.573**	0.022
Climatic Moisture Index	43	2	**0.906**	−0.107	**0.899**	−0.150
Continentality	49	8	**0.751**	**0.194**	**0.750**	**0.212**
Growing Deg Days 0C	35	6	**0.847**	0.048	**0.716**	0.046
Growing Deg Days 5C	48	3	**0.233**	−0.087	0.040	−0.076
Max Temp Coldest	19	0	**0.765**	−0.054	**0.758**	−0.068
PET Coldest Quarter	28	0	**0.742**	0.046	**0.781**	0.070

*Note:* Bold correlations are significant at *p* < 0.001.

The planted stands consistently show higher estimates of the risk of non‐adaptedness (RONA) across all climate variables we assessed, regardless of whether the RONAs were estimated using outlier loci selected based on allele frequency correlations or using LFMM (Figure 5A,B). The only cases in which RONA was not significantly greater in newly planted stands were DegD5, MTC, and PCQ for the LFMM‐based calculations (Figure 5B).

## 4 Discussion

We did not detect any systematic differences in the amount of genetic diversity maintained in old versus planted populations, regardless of what measure we used (Figure S3), although we do observe greater genetic differentiation between old‐growth and planted stands, and greater genetic similarities within planted stands across sites (Figure 3). These results together indicate that current forest management practices have not substantially reduced genetic variation at either the stand or the landscape level. These results are also in line with earlier studies that have assessed genetic diversity in forest trees and which have generally found no or only minor differences in estimates of genetic diversity between natural and managed stands of forest trees (Bergmann and Ruetz 1991; Garcia‐Gíl et al. 2015; Maghuly et al. 2006; Ruņģis et al. 2019; Sønstebø et al. 2018). Previous studies have used various genetic markers, including allozymes, microsatellites and RAPDs, but have largely failed to detect significant differences in genetic diversity between planted and natural stands. Bergmann and Ruetz (1991) used eight enzyme loci to compare 45 seed orchard trees with 60 random spruce trees from the same area and found a significant difference in average heterozygosity. Ruņģis et al. (2019) found higher numbers of alleles and higher average gene diversity in naturally regenerated forests (153 trees) in comparison to progeny from seed orchards (144 trees) when genotyping using 11 SSRs. Maghuly et al. (2006) also used SSRs, chloroplast SSRs and mitochondrial markers to compare two age classes and three populations at different elevations. Using 50 old and 100 young individuals from each population, they found no difference in seven chloroplast SSRs. Their five SSRs indicated that there was less genetic diversity and heterozygosity among the different populations than there was within populations. Sønstebø et al. (2018) compared two seed orchards (25 and 60 parents) with semi‐natural forests and unmanaged stands. Four samplings were conducted with 300 seeds each in the seed orchards, and 13 seed lots were collected from semi‐natural forests. Finally, needles were sampled from five natural forests. With 11 microsatellites, they showed a slightly lower genetic diversity in the form of allelic richness from the seed orchard samples, with the most pronounced difference seen in the orchard with only 25 parent trees (Sønstebø et al. 2018). One thing these studies have in common is that they have all relied on a relatively modest number of independent genetic markers, suggesting that the power to detect differences in genetic diversity has been low. Compared with earlier studies, our study uses a substantially larger number of independent genetic markers (~47 k SNPs) and also provides reasonable coverage of the spruce genome (> 8 k unique genomic regions). We have made an effort to sample from a relatively large geographic area in northern Sweden, spanning the Baltic coast to the Scandinavian mountains (~25,000 km^2^). We included 45 stands and a total of 1350 trees in our study, which, combined with a large number of markers, should provide substantially greater statistical power to detect differences in genetic diversity and differentiation. This allows us to confidently state that we do not observe any differences in genetic diversity between old and planted populations. If such differences nevertheless exist, they must be small. Although we cannot be certain of the origin of the seeds used for replanting trees at the planted stands in our study, statistics from the Swedish Forest Agency suggest that between 65% and 85% of all planted seedlings are derived from Swedish or foreign seed orchards (https://www.skogsstyrelsen.se/en/statistics/). Also, although seeds used to replant individual sites likely have a common origin (e.g., seed orchard or sampling site), different planted sites likely use material derived from vastly different sources. Since the planted populations were established between 1993 and 2008, any seed orchard material used for replanting is most likely derived from the second round of tree breeding (established 1981–1994) and is not representative of the most advanced selection made to date. It is possible that additional selection rounds could increase genetic diversity differences between natural and production forests, making them easier to detect; however, this remains to be investigated, for example by examining genetic diversity in seed lots sampled from operational seed orchards.

It is essential to distinguish between putatively neutral genetic variation and genetic variation explicitly linked to traits that confer an adaptive advantage and that mediate local adaptation. Population genetics theory suggests that the majority of the variation across a species' genome is likely neutral or nearly neutral, and only a small fraction of variants mediate adaptive traits (Charlesworth et al. 2017). We also know from many earlier studies that the genetic structure of adaptive variation is often fundamentally different from neutral genetic variation and that natural selection increases differentiation among populations for adaptive traits that contribute to local adaptation (Le Corre and Kremer 2003; Savolainen et al. 2013). Local adaptation persists in most forest trees despite high levels of gene flow that continuously introduces potentially maladaptive genetic variation (Kremer et al. 2012), and this is also the case in Norway spruce (Chen et al. 2012; Kapeller et al. 2017; Suvanto et al. 2016). Forest tree populations can adapt to local environments despite extensive gene flow, so natural selection acting on locally adaptive traits must be strong enough to overcome the homogenising effects of gene flow. So even if neutral markers display low genetic differentiation, consistent with extensive gene flow among populations, patterns of genetic differentiation at loci directly involved in controlling adaptive traits could be quite different, resulting in patterns of *isolation by environment* (IBE), where genetic and environmental distances are positively correlated, independent of their geographic distance (Wang and Bradburd 2014).

**FIGURE 4:**
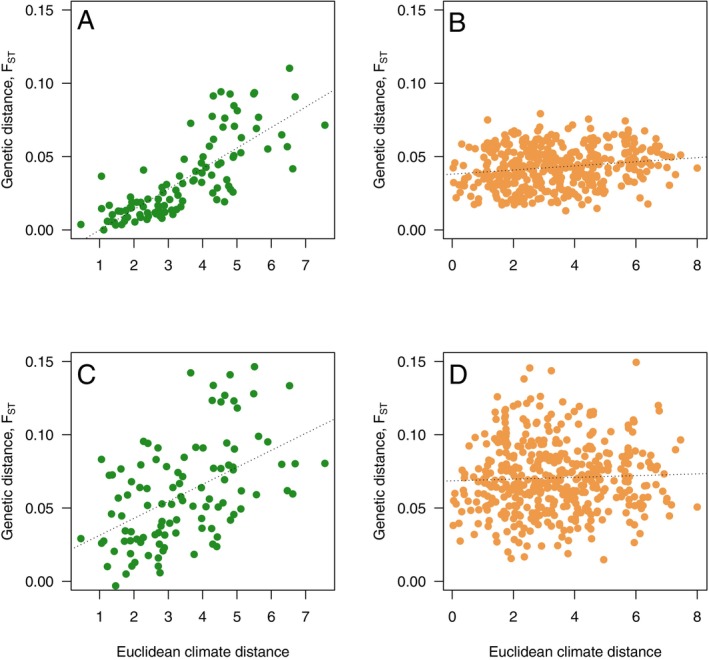
Associations between genetic distance and climate distance for the two set sof populations. Genetic distance is calculated from all SNPs that show a significant correlation with one or more climate variables. In (A) and (B) SNPs significant in the correlation analyses were used, whereas in (C) and (D) SNPs significant from the LFMM analyses were used. The dotted lines are the best‐fitting regression lines and are only used for visual guidance. In all comparisons, climate distance is calculated as the Euclidean distance between sites across all seven climate variables.

In line with these observations, a strikingly different picture emerges when we focus on adaptive genetic variation in the old‐growth and planted stands. The amount of variation and how it is associated with underlying climate variables differ drastically between the two types of forest stands we have studied (Figure 4, Tables 2 and 3). We observe strong and consistent correlations between genetic and environmental distance in the old‐growth populations (Figure 4, Tables 2 and 3), and this holds regardless of the method used to identify outlier loci assumed to represent adaptive variation. In contrast, we observe no or only weak correlations when assessing the same loci in the planted stands (Figure 4, Tables 2 and 3). We also observe a 10‐ to 20‐fold greater number of significant SNP‐climate associations in old‐growth forests compared to the planted populations (Tables 2 and 3). When we use these outlier SNPs to assess isolation by environment while simultaneously controlling for the background genetic structure, we consistently observe strong positive correlations in old‐growth forests (Tables 2 and 3, Figure 4). At the same time, this pattern is largely absent for the planted stands (Tables 2 and 3, Figure 4).

**FIGURE 5:**
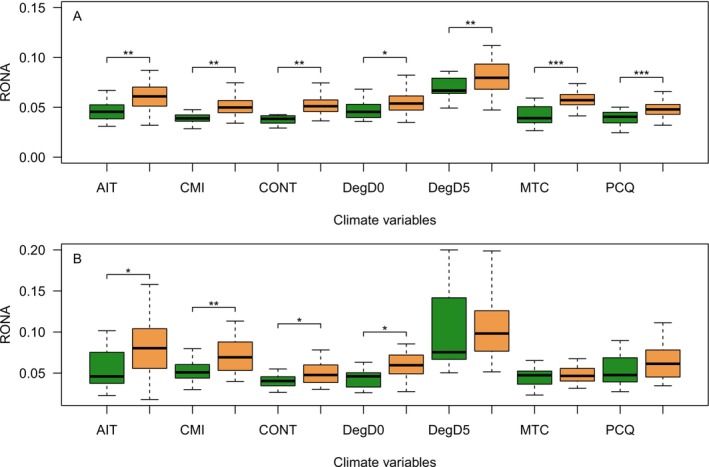
Risk of non‐adaptedness (RONA) for old‐growth (green) and newly planted (green) stands for all climate variables (see Table 1 for an explanation of abbreviations). In (A), outlier loci are identified using allele frequency correlations, and in (B), outlier loci are identified using LFMM.

When assessing the risk of non‐adaptedness (RONA) of current standing variation under a future climate warming scenario (RCP45), we observe that RONA estimates are consistently and significantly higher for the planted populations, suggesting these populations are at greater risk of suffering adverse effects from climate warming. However, it is worth emphasising that the higher RONA estimates in the planted populations partly reflect a mismatch already with the local climate under current conditions, as suggested by the limited evidence of local adsaptation we observe in these populations (Tables 2 and 3, Figure 4). It is therefore unclear whether these populations will suffer disproportionately from future climate change or whether their higher RONA value seen in planted populations (Figure 5) simply reflects this initial climate mismatch already present under the current climate.

Our results highlight a possible underappreciated consequence of large‐scale reforestation programmes in that they may weaken or disrupt patterns of local adaptation in managed populations planted when material used for replanting is derived from non‐local sources, such as seed orchards or seed collections from natural (but not necessarily local) populations. This suggests that planted populations could be exposed to greater risks from climate change if care is not taken to include local adaptation as a key criterion when selecting seed source material. Forest tree breeding is usually performed with local adaptation and future climate change in mind (Cortés et al. 2020). However, since the old‐growth populations we have assessed show substantially stronger associations with local climate than planted populations that were sampled from the same geographic areas, this suggests that old‐growth populations harbour important adaptive alleles that, for reasons unknown, are either absent or occur at low frequencies in source populations used for reforestation of the planted stands. Old‐growth populations may thus serve as important sources for climate‐adapted alleles, and future work should focus on identifying these alleles and making a dedicated effort to introduce them into breeding populations and relevant seed orchards used in reforestation.

An important caveat of climate‐vulnerability estimates based on allele frequency differences across environments and clinal variation is that such variation only serves as proxies for potential adaptive mismatches, rather than directly assessing their fitness effects in controlled field or common garden experiments (Lind et al. 2024; Lotterhos 2024). Climate‐vulnerability estimates, such as RONA, rely on the degree of genetic change needed for a population to track and adapt to future climate conditions. Still, they do not directly measure the actual reproductive success, survival, or overall fitness of individuals in a changing environment (Lind et al. 2024; Lind and Lotterhos 2024; Lotterhos 2024). Although allele‐frequency‐based estimates have been shown to correlate with fitness declines in controlled experiments, such as common garden studies, they do not inherently account for other ecological factors that influence fitness in natural settings, making them proxies rather than direct measures of maladaptation or vulnerability (Lind and Lotterhos 2024; Villoutreix et al. 2025).

## 5 Conclusions

This study provides a comprehensive comparison of genetic diversity and adaptive potential between old‐growth and recently planted stands of Norway spruce ( *Picea abies* ) in northern Sweden. Utilising extensive genomic data and broad geographic sampling, our results reveal no significant differences in neutral genetic diversity between natural and planted populations, suggesting that current forestry practices, including seed orchard selections and reforestation methods, have not substantially impacted overall genetic variation at either the stand or landscape levels. However, the patterns are strikingly different when assessing adaptive variation that contributes to large‐scale climate gradients, variation that is also crucial for predicting future responses to climate change. Old‐growth forests show substantially stronger patterns of local adaptation and are also predicted to face lower risks from future climate change. These findings underscore the importance of maintaining and enhancing adaptive genetic diversity through forest management to ensure resilience to future climate change. While planting genetically improved seedlings from seed orchards does not diminish overall genetic diversity in Norway spruce populations and helps accelerate reforestation, careful consideration is necessary to preserve genetic variation essential for local adaptation. Old‐growth forests could serve as important sources for (re‐)introducing such adaptive variation into existing tree breeding programmes.

## Funding

This work was supported by Knut och Alice Wallenbergs Stiftelse and Stiftelsen för Strategisk Forskning (RBP14‐0040).

## Conflicts of Interest

The authors declare no conflicts of interest.

## Supporting information


**Table S1:** Type, location and age of sampling sites and summary statistics of sequencing data per library.
**Table S2:** Summary statistics of the sequencing data.
**Figure S1:** Summary of the ENVIREM climate variables for all populations used in the study. The diagonal displays the distribution of the individual variables, figures below the diagonal display pairwise scatterplots for all variables and above the diagonal, the corresponding correlation coefficients are given. *p < 0.05, **p < 0.01, ***p < 0.001.
**Figure S2:** (A) Principal component analysis and (B) screeplot of the allele frequency data. Old and planted populations in (A) are depicted using green and tan, respectively.
**Figure S3:** (A) The number of invariable sites, (B) the proportion of rare alleles (alleles with frequency < 0.05) and (C) heterozygosity in planted (tan) and old (green) populations. None of the population comparisons are significantly different.

## Data Availability

The GBS raw reads have been deposited in NCBI's Sequence Read Archive (SRA) under accession number PRJEB89643 (https://www.ncbi.nlm.nih.gov/bioproject/PRJEB89643/). Background information on all sites, environmental data at the site of origin for all populations and the PoolSeq SNP data are available from Zendo (https://doi.org/10.5281/zenodo.16880968) under a CC BY‐SA 4.0 license. All scripts used for the analyses described in the paper are available on GitHub under a MIT License (https://github.com/parkingvarsson/SpruceOldvsPlanted).
