# Supplementary material for: Contrasting Patterns of Local Adaptation and Adaptive Potential Under Climate Change for Old‐Growth and Planted Stands of Norway Spruce (Picea abies)
Source: Evol Appl. 2026 Mar 12;19(3):e70217. doi: 10.1111/eva.70217 (PMC13093344; doi:10.1111/eva.70217)
Supplement: Supplementary file 1 — Table S1: Type, location and age of sampling sites and summary statistics of sequencing data per library. Table S2: Summary statistics of the sequencing data. Figure S1: Summary of the ENVIREM climate variables for all populations used in the study. The diagonal displays the distribution of the individual variables, figures below the diagonal display pairwise scatterplots for all variables and above the diagonal, the corresponding correlation coefficients are given. *p < 0.05, **p < 0.01, ***p < 0.001. Figure S2: (A) Principal component analysis and (B) screeplot of the allele frequency data. Old and planted populations in (A) are depicted using green and tan, respectively. Figure S3: (A) The number of invariable sites, (B) the proportion of rare alleles (alleles with frequency < 0.05) and (C) heterozygosity in planted (tan) and old (green) populations. None of the population comparisons are significantly different. [file EVA-19-e70217-s001.pdf]

Supplementary Table S1. Type, location and age of sampling sites and summary statistics of sequencing data per library.

Area	Number	Type	Latitude	Longitude	Altitude (m)	Company	Age	SweRef.X	SweRef.Y
Kålhuvudet	P1.1	Old	63.63	18.46	352	-	-	7060749	671479
	P1.2	Planted	63.67	19.14	154	SCA	23	7066643	704930
	P1.3	Planted	63.66	19.16	144	SCA	16	7066039	705714
Långrumpskogen	P2.1	Old	63.69	19.54	106	-	-	7070934	724401
	P2.2	Planted	63.67	19.52	121	SCA	16	7068243	723552
	P2.3	Planted	63.70	19.36	135	SCA	14	7071308	715622
Balberget	P3.1	Old	63.94	19.12	308	-	-	7097346	701969
	P3.2	Planted	64.06	18.50	322	Sveaskog	14	7108319	670875
	P3.3	Planted	64.05	18.54	330	Sveaskog	18	7107804	672950
Stenbithöjden	P4.1	Old	64.04	16.91	520	-	-	7102419	593220
	P4.2	Planted	64.02	16.99	381	Sveaskog	19	7100918	597489
	P4.3	Planted	64.03	16.93	463	Sveaskog	19	7101722	594314
Nordansjöberget	P5.1	Old	64.11	17.83	508	-	-	7112743	637883
	P5.2	Planted	64.14	17.84	363	Sveaskog	20	7115590	638267
	P5.3	Planted	64.10	17.76	439	Sveaskog	9	7111391	634433
Fjällheden	P6.1	Old	64.15	18.69	411	-	-	7119021	679619
	P6.2	Planted	64.15	18.76	323	Sveaskog	18	7119139	682909
	P6.3	Planted	64.15	18.67	434	Sveaskog	20	7119375	678469
Järvtjärn	P7.1	Old	64.22	19.26	322	-	-	7128561	706539
	P7.2	Planted	64.20	19.18	309	Sveaskog	12	7125515	702785
	P7.3	Planted	64.08	19.40	234	SCA	18	7113267	714357
Skikki	P8.1	Old	65.03	16.54	648	-	-	7212748	572376
	P8.2	Planted	65.00	16.59	494	SCA	16	7209820	574870
	P8.3	Planted	65.00	16.63	483	SCA	15	7209310	577045
Arasjö	P9.1	Old	64.55	17.61	524	-	-	7161212	625306

	P9.2	Planted	64.50	17.49	473	SCA	16	7154801	619544
	P9.3	Planted	64.50	17.51	506	SCA	21	7155503	620687
Altarliden	P10.1	Old	64.79	18.82	358	-	-	7190411	681710
	P10.2	Planted	64.80	18.83	273	Sveaskog	16	7191313	681861
	P10.3	Planted	64.79	18.85	332	Sveaskog	19	7190643	682692
Vitberget	P11.1	Old	64.63	19.73	430	-	-	7175481	725771
	P11.2	Planted	64.77	19.50	325	Sveaskog	19	7190573	713790
	P11.3	Planted	64.76	19.52	293	Sveaskog	13	7189647	714680
Marsfjället	P12.1	Old	65.09	15.86	604	-	-	7218574	540454
	P12.2	Planted	64.88	15.81	512	SCA	24	7195198	538373
	P12.3	Planted	64.85	15.80	491	SCA	19	7191909	537826
Kirjesålandet	P13.1	Old	65.57	16.11	526	-	-	7272926	551422
	P13.2	Planted	65.31	16.80	551	Sveaskog	17	7243901	583667
	P13.3	Planted	65.29	16.82	511	Sveaskog	23	7242554	584875
Nalovardo	P14.1	Old	65.68	17.56	666	-	-	7286213	617439
	P14.2	Planted	65.65	17.59	431	Sveaskog	10	7283266	619120
	P14.3	Planted	65.64	17.57	458	Sveaskog	10	7282524	618385
Granliden	P15.1	Old	65.48	18.21	452	-	-	7265511	648682
	P15.2	Planted	65.52	17.99	414	Sveaskog	14	7269599	638131
	P15.3	Planted	65.50	18.00	444	Sveaskog	15	7267612	638978

Supplementary Table S2. Summary statistics of the sequencing data.

**Number of unique regions**	8731
**Total number of sites covered (bp)**	1,172,909
**Coding sites (%)**	20.80%
**Repeats (%)**	19.00%
**Mean size of GBS regions (bp)**	140
**Total number of SNPs**	47,552

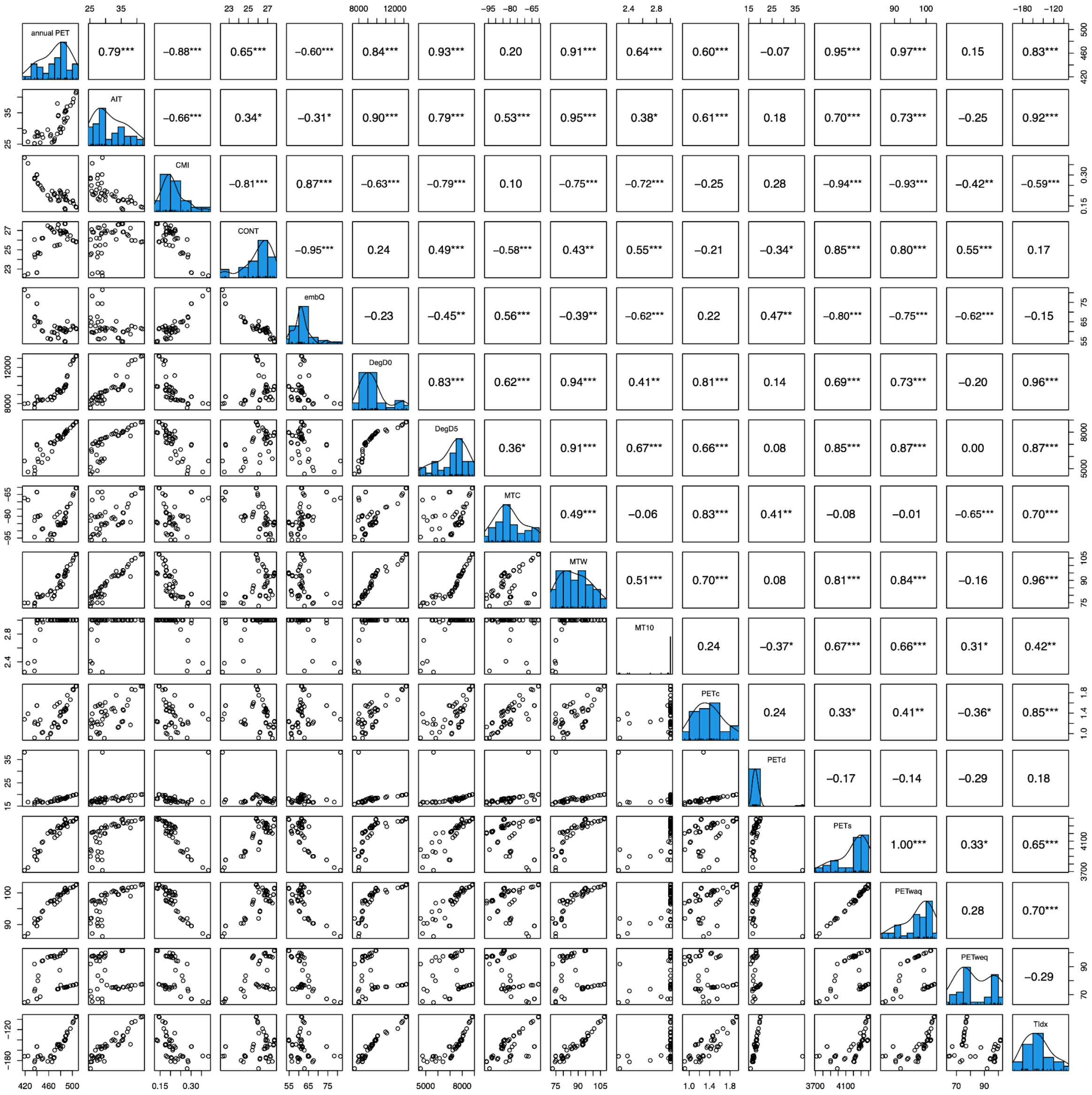

annual PET
AIT
CMI
CONT
embQ
DegD0
DegD5
MTC
MTW
MT10
PETc
PETd
PETs
PETwaq
PETweq
TIdx

Supplementary Figure S1. Summary of the ENVIREM climate variables for all populations used in the study. The diagonal displays the distribution of the individual variables, figures below the diagonal display pairwise scatterplots for all variables and above the diagonal, the corresponding correlation coefficients are given. *$p<0.05$, **$p<0.01$, ***$p<0.001$

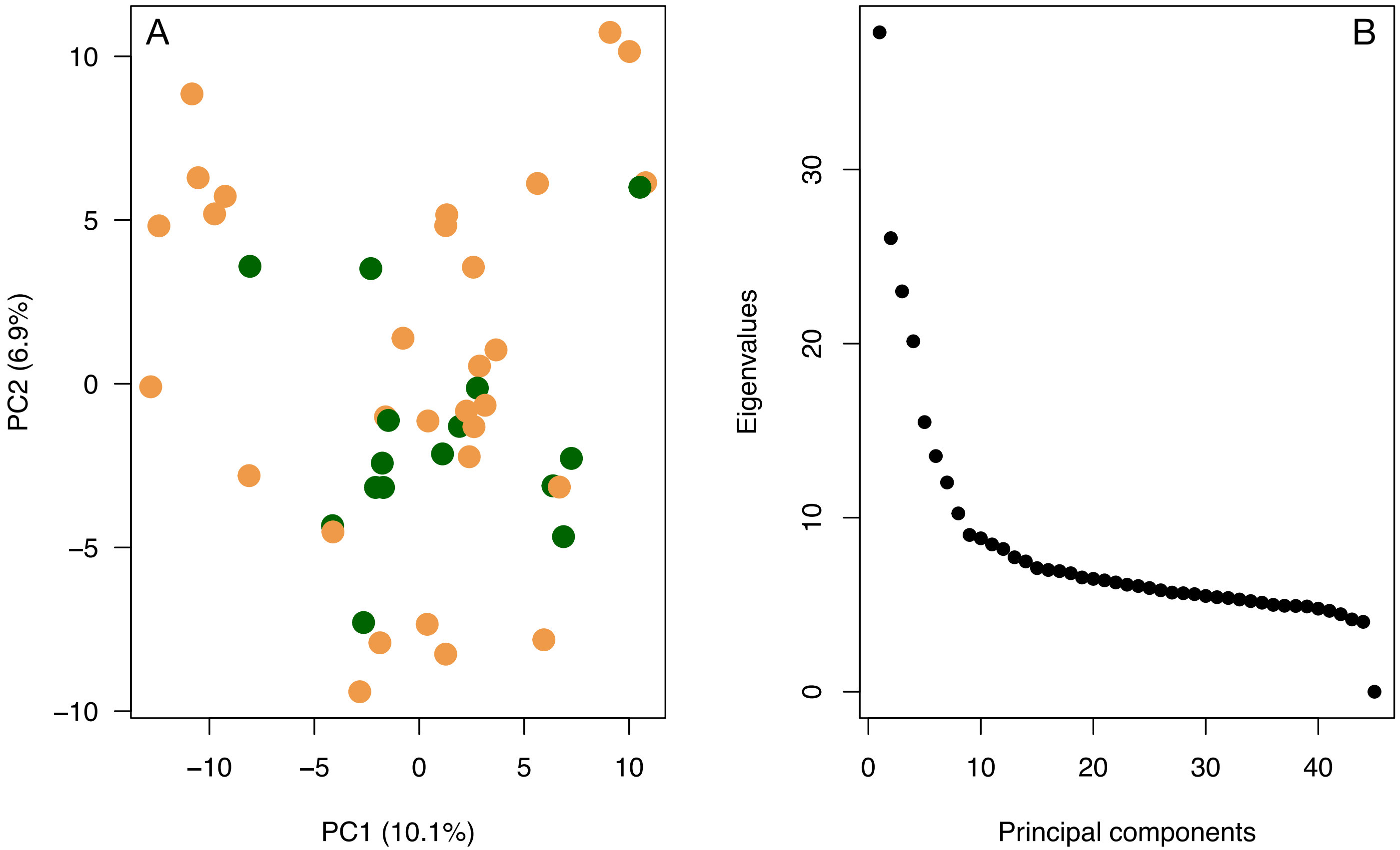

Supplementary Figure S2. A) Principal component analysis and B) screeplot of the allele frequency data. Old and planted populations in A) are depicted using green and tan, respectively.

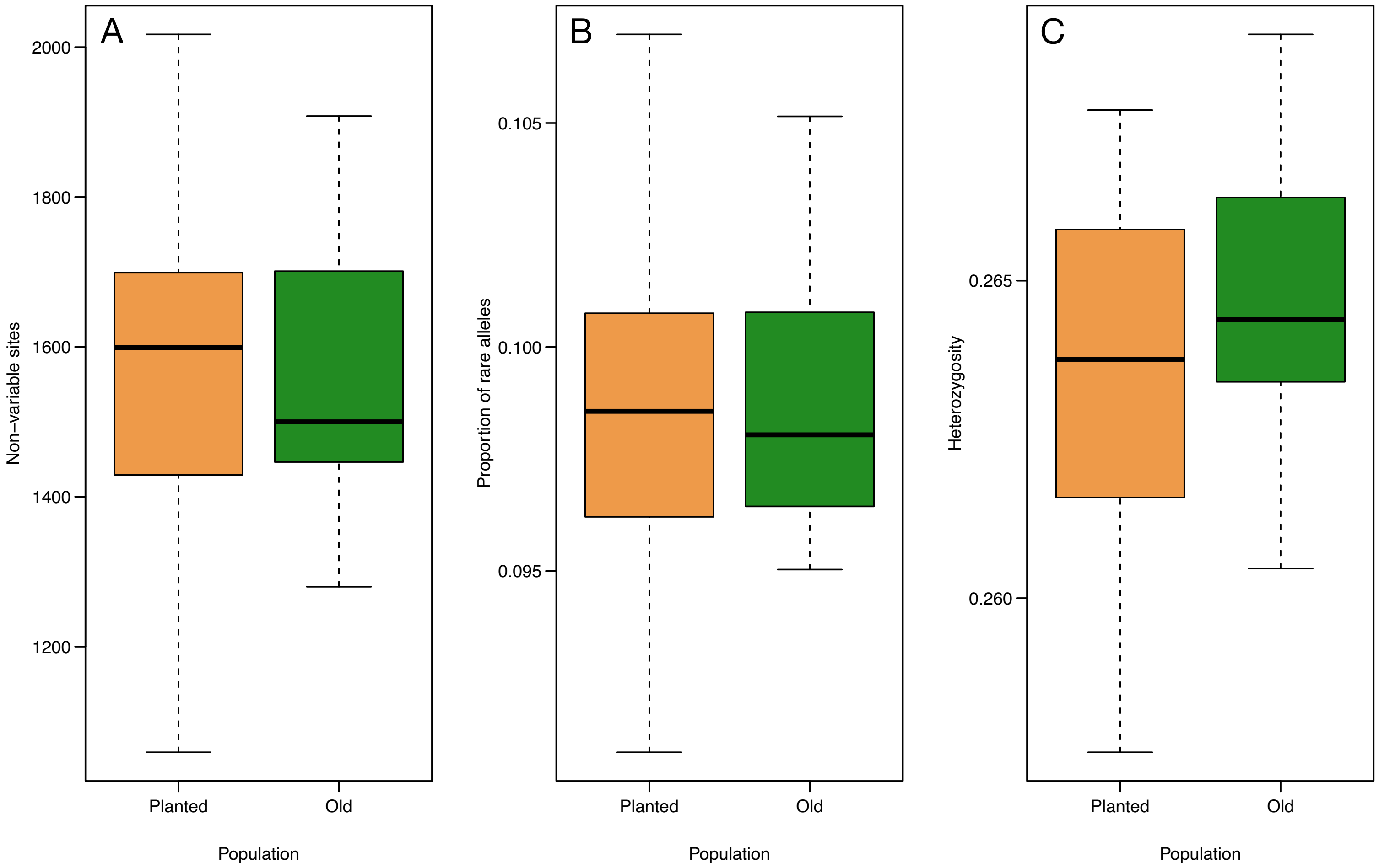
A
B
C
Non−variable sites
2000
1800
1600
1400
1200
Proportion of rare alleles
0.105
0.100
0.095
Heterozygosity
0.265
0.260
Planted
Old
Population

Supplementary Figure S3. A) The number of invariable sites, B) the proportion of rare alleles (alleles with frequency <0.05) and C) heterozygosity in planted (tan) and old (green) populations. None of the population comparisons are significantly different.
